# Identifying the social and environmental determinants of plague endemicity in Peru: insights from a case study in Ascope, La Libertad

**DOI:** 10.1186/s12889-018-5062-0

**Published:** 2018-02-06

**Authors:** Ana Rivière-Cinnamond, Alain Santandreu, Anita Luján, Frederic Mertens, John Omar Espinoza, Yesenia Carpio, Johnny Bravo, Jean-Marc Gabastou

**Affiliations:** 1 Pan-American Health Organization/World Health Organization (PAHO/WHO), PAHO Health Emergencies Department (PHE), Los Pinos 251, Camacho La Molina, 12 Lima, Peru; 2 Consorcio por la Salud, Ambiente y Desarrollo (ECOSAD), Lima, Peru; 3 Centro de Desenvolvimento Sustentável, Universidade de Brasília, Brasília, Brazil

**Keywords:** Plague, Social and environmental determinants of health, Qualitative methods, Risk perceptions, Complex systems, Public health policy

## Abstract

**Background:**

Plague remains a public health problem in specific areas located in Bolivia, Brazil, Ecuador and Peru. Its prevention and control encompasses adequate clinical management and timely laboratory diagnosis. However, understanding communities’ interaction with its surrounding ecosystem as well as the differences between community members and institutional stakeholders regarding the root causes of plague might contribute to understand its endemicity. We aim at bridging the traditionally separate biological and social sciences by elucidating communities’ risk perception and identifying knowledge gaps between communities and stakeholders. This approach has been used in other areas but never in understanding plague endemicity, nor applied in the Latin American plague context. The objectives were to identify (i) plague risk perception at community level, (ii) perceived social and environmental determinants of plague endemicity, and (iii) institutions that need to be involved and actions needed to be taken as proposed by stakeholders and community members. The study was performed in 2015 and took place in Ascope rural province, La Libertad Region, in Peru, where the study areas are surrounded by intensive private sugarcane production.

**Methods:**

We propose using a multi-level discourse analysis. Community households were randomly selected (*n* = 68). Structured and semi-structured questionnaires were applied. A stakeholder analysis was used to identify policy makers (*n* = 34). In-depth interviews were performed, recorded and transcribed. Descriptive variables were analyzed with SPSS®. Answers were coded following variables adapted from the Commission on Social Determinants of Health and analyzed with the assistance of ATLAS.ti®.

**Results:**

Results showed that risk perception was low within the community. Policy-makers identified agriculture and sugarcane production as the root cause while community answers ranked the hygiene situation as the main cause. Stakeholders first ranked governmental sectors (education, housing, agriculture and transport) and the community prioritized the health sector. Social surveillance and improving prevention and control were first cited by policy-makers and community members, respectively.

**Conclusions:**

The determinants of plague endemicity identified by the two groups differed. Similarly, actions and sectors needed to be involved in solving the problem varied. The gaps in understanding plague root causes between these two groups might hinder the efficiency of current plague prevention and control strategies.

## Background

Plague is still endemic and widespread in many low-income countries [1, 2]. Although middle income countries have managed in most instances to control the disease, plague episodes may occur after long epidemiologically silent periods (e.g. over 50 years) [3–5].

In Latin America, despite its enhanced control in the past decades, plague continues to be endemic in specific hotspot areas [6]. From 2000 to date, the productive hotspots have been identified in Franz Tamayo and Andres Ibañez (Bolivia), Feira de Santana and Pedra Branca (Brazil), Guamote, Riobamba, Latacunga (Ecuador) and Chota, Contumaza, Cutervo, Jaen, San Miguel, Santa Cruz, Ascope, Otuzco, Pacasmayo, Trujillo and Ferrenafe (Peru) and their ecosystems characterized in detail [1].

Plague has three clinical presentations: bubonic, septicemic and pneumonic. It is caused by the telluric bacteria *Yersinia pestis*. The reservoirs are wild rodents for the sylvatic cycle and synantropic rodents such as *Rattus rattus* or *Rattus norvegicus* for the peridomestic cycle. It is transmitted through flea bites (the vector), particularly *Xenopsila cheopis*. Rodents, either wild or peridomestic, get infected through flea bites and develop the disease. An unusually high number of infected animals within a specific period of time in a region is defined as an epizootic. As observed in other zoonoses, epizootics are often followed by outbreaks of human cases. Indeed, as rodents die of plague, fleas carry *Yersinia pestis* during their search for other hosts. Other animals such as cats [7] and guinea pigs [8, 9] have been reported to have a role in plague transmission. Guinea pigs (*Cavia porcellus*) do also get infected and die of plague. In the rural Andean culture, they play a key role in plague transmission to humans since they are an important part of the diet and are kept within or near the household. Decision-makers or stakeholders in health and other sectors in Peru tend to be aware of the risks associated with such cultural practice. However community members tend to be reluctant to change their behavior [8]. Such reluctance might be linked to community members’ low risk perception levels regarding plague. The different perceptions regarding the causative determinants of plague between these two groups of actors (i.e. stakeholders and community members) may affect the implementation of integrated solutions. Hence the idea of identifying the risk perceived at local level regarding plague and analyzing and comparing between community and stakeholders their perception of what are the determinants of plague, which sectors should be involved in solving the problem and the solutions the two groups propose. The gaps between these two groups might help in understanding the need for stronger collaborative processes between communities and stakeholders for plague prevention. Several authors have engaged in this type of comparison between groups albeit in other fields. Masozera studied the perceptions between policy makers and community members regarding a community-based management (CBM) approach focusing on Rwandan Nyungwe Forest Reserve. Their study highlighted the opposing views over the CBM between the groups, hence the need for closing the gap between these two groups regarding the understanding of the CBM approach [10]. Similarly, Ruebush compared the perceptions of community and policy makers associated with the “ideal volunteer malaria worker” in Guatemala [11]. They found that the disparities between the two groups might hinder the sustainability of malaria control strategies at the community level. Similar results regarding community animal health workers have been found in other settings such as Kenya [12] whereby diverging perception of qualities in community workers between groups hindered the sustainably of the community-based scheme.

Plague prevention and control encompass three main components: (i) clinical and epidemiological aspects; (ii) a laboratory component, including early detection, monitoring, surveillance and strain characterization; and (iii) the social and environmental determinants of plague. Efforts have greatly improved the first two components in terms of event and case management protocols [13, 14], disease epidemiology [15, 16], laboratory diagnostic and molecular techniques [17]. However, in spite of all such improvements, the aforementioned hotspots continue to be endemic, producing cases on a yearly basis [2].

Environmental and social or behavioral factors are commonly cited in peer reviewed articles as having an impact in plague emergence [18–23]. On the environmental side, several studies have identified the ranges of altitude, rainfall, biomes and soil types associated with the remaining plague endemic areas in Bolivia, Brazil, Ecuador and Peru [1]. Other studies in Latin America and elsewhere have also addressed several socio-environmental risk factors for plague occurrence. These included landscape features, ecotones and elevation, but interestingly also other variables related to social determinants and human behavior such as distance to water, [24], sleeping mats materials, food storage, livestock and pet ownership, home maintenance, hut structure, types of crops, rodent and flea control strategies [8, 25]. Climatic variables and El Niño Southern Oscillation (ENSO) events have also been linked to plague resurgence [26–28]. This follows the lines of McMillan [19] who emphasized that in similar ecological or environmental conditions, plague occurrence was highly related to social determinants and human behavioral factors.

Understanding the social component associated with plague in endemic areas is crucial to comprehend human interaction with its surrounding ecosystem and how it may contribute to diseases emergence [29, 30]. Several authors have coined different terms to address this concept: “biocomplexity paradigm” [31]; “social-ecological systems” [32, 33]; or “human and natural systems” [34]. They all highlight the importance of the “interaction of humans and nature as a complex system” [29] and propound the idea of generating a “social-ecological approach for addressing and garnering and improving understanding of emerging infectious diseases” [29]. Indeed ecological factors affect infectious disease emergence or re-emergence. But “the scale and magnitude of anthropogenic activity has reached a point of virtual co-dominance with natural processes” [30]. Parham highlights that understanding the complexity of human society requires novel scientific approaches to identify fundamental drivers’ dynamics and to facilitate interventions which need to be supported by appropriate policies [35]. Despite extensive work performed on plague on biological disciplines and others, little has been performed to explore human behavior and risk perceptions and their link to ecological changes and disease emergence.

Social drivers or determinants have been long been pointed out as crucial in addressing infectious diseases [36, 37]. Farmer mentioned in 1996 that “Ebola, TB, and HIV infection are in no way unique in demanding contextualization through social science approaches. These approaches include the grounding of case histories and local epidemics in the larger biosocial systems in which they take shape” [36]. Hence, social sciences and related analytical tools have been used in addressing infectious diseases. A notable example is the case of HIV/AIDS. Auerbach highlighted that despite the increased knowledge on the biology of HIV, for effectively curbing the epidemic, social drivers should be addressed [38]. Interestingly, they mention that “Social/structural approaches aim to modify social conditions and arrangements by addressing the key drivers of HIV vulnerability that affect the ability of individuals to protect themselves and others from acquiring or transmitting HIV infection” [38]. In this quote, the term “HIV” could perfectly be changed by “plague” and the sense will still remain and be applicable.

With this study we aim at bridging “theory from the traditionally separate biological and social science disciplines” [30]. To date, social sciences analytical techniques have neither been used in understanding plague endemicity, nor applied in the Latin American plague context.

The 2010 plague outbreak in La Libertad, Peru, where 27 cases were detected and 4 deaths occurred [2], led the Pan-American Health Organization (PAHO/WHO) to rethink the regional plague prevention and control strategy. A “Strategic Plan for Integrated Surveillance and Control of Plague in South America” (thereafter, the Plan) was elaborated, its main objective being the one stated by the PAHO Governing Bodies Resolution CD49/R19 [39] of (i) zero human death and (ii) zero intra-domiciliary cases. The Plan was grounded on the 3 main strategic axes mentioned earlier. The two first axes are being dealt with. However, to date no structured effort has been devised to translate the social and environmental determinants at local level into a tangible tool and a specific methodology.

Social and environmental determinants of health [40–43] are increasingly being mentioned as the root causes of many infectious diseases [23, 30]. They represent an important section in every disease control strategy stated by PAHO/WHO. In the specific case of plague, several environmental and ecological determinants have been identified as risk factors for disease occurrence. These include: landscape features, elevation, distance to water, ecotones [24], sleeping mats materials, food storage, livestock and pet ownership, home maintenance, hut structure, types of crops, rodent and flea control strategies [8, 25]. Climatic variables and El Niño Southern Oscillation (ENSO) events have also been linked to plague resurgence [26–28]. However, the only behavioral factors found to date to have been included in a case-control study performed in Uganda were “health-care seeking” and “level of knowledge” regarding plague [25]. For example, the results of this study point out that case location respondents had a significant tendency of seeking treatment from traditional healers instead of going to healthcare centers. These results illustrate the importance of understanding and including behavioral factors when elaborating disease control strategies, particularly early detection and reporting.

In this study we consider that (i) the social aspects at local level, including culture, perceptions and beliefs regarding plague are highly entangled with the ecosystem or environmental conditions where people live, and that (ii) adding a structured extra layer of qualitative information to the traditionally only quantitative approach to plague prevention and control might help in understanding plague endemicity and emergence. We engage in a comparison between two groups: local inhabitants at community level and decision-makers or stakeholders involved in plague response at community, local and regional levels, to identify any differences in ranking determinants of plague and actions suggested.

We propose the use of discourse analysis in a multi-level approach to identify (i) plague risk perception at community level, (ii) the perceived social and ecological determinants of plague for community members as well as for decision-makers, and (iii) the actions proposed by these two groups that would facilitate preventing plague occurrence. The study was performed in 2015 and took place in the province of Ascope, Region of La Libertad, in Peru.

## Methods

### Study location

For studies such as this, the rural setting represents the social conditions in which the local population lives and crop production, which has been linked to increased rodent presence, can be assessed as illustrating the environmental determinants [44]. Considering these elements, the criteria for selecting the study location were: (i) being in a rural setting with intensive agricultural production; (ii) being under the jurisdiction of a single Primary Health Center of the Ministry of Health; (iii) availability of epidemiological data on *Yersinia pestis* circulation or human confirmed plague cases between November 2013 and September 2014; (iv) security and accessibility; and (v) explicit support from the regional authorities as well as from the local health and non-health actors.

The study locations selected were the rural areas of “Los Colonos”, “Santa Clara” and “San José Alto” with 50, 140 and 10 households respectively (source: Regional Health Authority of La Libertad or “Gerencia Regional de Salud La Libertad” - GERESA LL- in Spanish), within the rural district of Casa Grande, province of Ascope, in the region of La Libertad, Peru. The district is located 29 km north of the city of Trujillo, the capital of the La Libertad region. The total population of the study area was 914 inhabitants served by the Casagrande Healthcare Center. The rural areas were surrounded by intensive sugarcane production managed by the private companies Cartavio and Casagrande. The total area of intensive sugarcane production in La Libertad region is of 37.067 ha [45].

Epidemiological data regarding circulation of *Yersinia pestis* and confirmed human plague cases for the study period was provided by national and GERESA LL, who fully supported the purpose of the study (Table 1).

**Table 1:** Yersinia pestis reservoir circulation surveillance in 2014 (source: National Institute of Health of Peru and GERESA La Libertad)

*Y. pestis reservoir surveillance in Santa Clara and Los Colonos, Casagrande*							
					*X. cheopis Specific Index (* ^*c*^ *)*		
Location	Num. traps	Captured rodents	Trap Index (^a^)	General Flea Index (^b^)	*R. rattus*	*R. norvegicus*	*Y. pestis* results
Los Colonos	60	17	14,17%	6,59	11,8	0,5	Not available
Santa Clara	60	12	10,00%	0,20	0	0,11	POSITIVE

(Note: No data was available for San José Alto but the human cases where located there)

(^a^) Defined as the number of traps with rats divided by the total number of traps deployed, multiplied by 100. [50]

(^b^) Defined as the number of fleas collected divided by the total number of rodents captured. [50]

(^c^) Defined as the number of fleas of one specific species divided by the total number of rodents of one specific species. If the Specific Index (SI) is > 1 regarding Xenopsilla cheopis, the situation is considered at risk. [50]

In addition there were 2 confirmed plague cases – one bubonic (19 years old male) and one septicemic (17 years old male) – during the epidemiological week 32 of 2014 (confirmation by the National Institute of Health of Peru) in San José Alto.

### Population (including sample definition)

#### Community level

Households were chosen as units of analysis. The 200 households located in the rural settings of Los Colonos, Santa Clara and San José Alto were identified as the study area. A representative sample size was calculated with 95% CI, *p* < 0.5 and s.d. = 10%. Sampling was stratified and considered each locality as one stratum. Sample units were selected randomly through a random numbers table. The observational unit was determined to be the adults present in each household during the visit, hence *n* = 68.

#### Decision-makers

A stakeholder analysis was elaborated through a participatory workshop. Stakeholders were identified and selected through a snow-ball technique until saturation. They represent the decision-makers from the sectors that were involved in the response in the November 2013–September 2014 plague outbreak in the locality. A total number of 24 stakeholders from different sectors were identified. During the workshop, stakeholders self-classified themselves into public, private and civil society sectors. In addition, level of involvement in outbreak response was defined as “directly involved” or, “indirectly involved”. Stakeholders’ administrative level of contribution was also defined: local, district/provincial, regional and national levels. During the workshop, 10 additional stakeholders that were “not involved but should have been involved” – according to the participants’ perceptions - in the plague outbreak response were mentioned by the participants and included in the mapping exercise. The total number of the decision-makers’ sample was *n* = 34.

### Data collection

#### Community members

Data was gathered through a questionnaire, which had been pretested and was divided in two sections. The first section was structured to collect socio-demographic and behavioral data, including age, gender, education level, household infrastructure (walls, roofs, floors), availability of utilities (water, sanitation, solid waste), food storage, livestock and pet ownership and keeping practices, and frequency of seeing rats and fleas. The second section consisted of a semi-structured questionnaire aimed at gathering information on determinants and risk perception. Questions focused on knowledge of plague (such as: symptoms, vector, reservoir, transmission mechanisms and health seeking behavior) and on the causes that facilitate plague endemicity and occurrence (such as: What are the root causes of plague?, Who is/are responsible of the existence of plague?, Who should be involved in plague prevention?, What actions should be implemented to solve the situation?). The purpose of the study was mentioned in every interview. The answers were hand written in a printed copy of the questionnaire. Data collected were transcribed for analysis into an Excel spreadsheet.

#### Decision-makers

Individual in-depth interviews were performed with the decision-makers group identified through the stakeholder analysis. Interviews were recorded and transcribed in Word document.

### Data analysis

#### Quantitative data

Descriptive data was analyzed with SPSS®. Complex sample analysis was used to devise homogeneity and representativity of the sample.

#### Qualitative data

Data obtained from the in-depth interviews and semi-structured interviews were analyzed with the assistance of ATLAS.ti® following a coding process. Variables for coding derived from Commission on Social Determinants of Health report [43]. The applicable variables which represented the social and environmental determinants in the Ascope local setting were: (i) agricultural activities, (ii) infrastructure and basic services, (iii) local culture, (iv) poverty, (v) local governance, (vi) sanitary situation. Words and concepts included in the transcribed interviews were coded. Each coded word or concept was then associated to one of the abovementioned variables with the assistance of ATLAS.ti through an iterative process.

### Ethical considerations

The research protocol was presented to the authorities of the National Zoonoses Strategy (“Estrategia Nacional de Zoonosis – ESNZ” in Spanish) of the Peruvian Ministry of Health, which is the unit in charge of plague. The protocol was accepted by the authorities. Since there is no formal ethics committee within the current Ministry of Health, and the survey did not collect information related to participants’ personal medical history or data, ethical approval was not required. However, all participants were read the informed consent which was digitally recorded. No remuneration was provided for participating in the study.

## Results

### Sample description

The majority of the community sample was composed of women (74%) mostly aged between 34 and 54 (53%) with high school educational level (52%), born in La Libertad (83%) and with permanent residency in the study rural annexes, largely Santa Clara (69%) and Los Colonos (25%). Main activity was house-work (59%). Other activities include selling, agriculture, and petty commerce.

### Living conditions at community level

It is important to highlight that the participants interviewed were living in households surrounded by intensive sugarcane productions. This is significant because of the epidemiological links between the peridomestic synantropic rodents’ ecological cycle, rodents’ dynamics and the possibility of interaction between rodents and household members. Local living conditions are summarized in the following (Table 2).

**Table 2:** Summary of descriptive data of local living conditions

Categories	Percentage	Confidence Interval 95%		Standard deviation	Sample (n)	Population
		Inferior	Superior			
Electricity supply						
24 h	79.1	71.9%	84.8%	4.1	54	158
No supply	20.9	15.2%	28.1%	15.5	14	42
Water supply						
1 h	1.5	0.2%	10.1%	100.0	1	3
6 h	77.6	69.7%	83.9%	4.6	53	155
24 h	5.9	2.3%	14.7%	47.4	4	12
No supply	15.0	9.2%	23.5%	23.6	10	30
Excreta disposal						
24 h	76.0	68.6%	82.2%	4.5	52	152
No disposal	24.0	17.8%	31.4%	14.2	16	48
Solid waste removal						
Up to 2 weeks	55.6	44.3%	66.4%	10.1	38	111
3 weeks/ No removal	44.4	33.6%	55.7%	12.7	30	89
Walls (materials)						
Adobe	56.4	45.4%	66.7%	9.6	38	113
Cement	3.0	0.7%	11,8%	70.8	2	6
Brick	40.6	30.8%	51.3%	12.8	28	81
Floors (materials)						
Cement	41.4	30.2%	53.4%	14.3	28	83
Stone/ mud	4.4	1.4%	13.0%	56.5	3	9
Fake floor	22.2	13.7%	33.9%	22.9	15	44
Soil	32.1	22.0%	44.2%	17.6	22	64
Roofs (materials)						
Corrugate/ Eternit	44.1	32.6%	56.2%	13.7	30	88
Cement/ Brick	33.5	24.2%	44.4%	15.2	23	67
Rush mat/ wattle and daub/ other	22.4	14.0%	33.8%	22.2	15	45

Most households had adobe walls, cement floor, did not have continuous running water, and did not have frequent solid waste removal.

41% of the study population saw rodents frequently (monthly/weekly/daily), 59% occasionally during the year and 76% rarely saw fleas. 60% had cats or dogs in their household and 29% stated having guinea pigs inside the household.

### Risk perception at community level

Results from the semi-structured interviews showed that 91% had “heard about plague” and indicated that it was considered a problem because it generated health consequences (51%) and could cause death (18%). Regarding risk perception, community members believed there was a strong association between rodents and health-risks (97%) and a less strong association between rodents and plague (47%). 58.3% did not know the transmission mechanisms of plague and 33.9% were not aware of the symptoms of plague.

### Stakeholder analysis

The institutional stakeholder analysis included 34 persons. The 24 participants who were initially selected mentioned 10 additional stakeholders who should have been involved in plague prevention and control but were not. Of the first 24 interviewed, 11 came from the health sector, four from local or district governments and one from the education sector. The 11 stakeholders from the health sector were directly involved in plague prevention and control (i.e. were part of the intervention and/or planning activities). Four non-health sector stakeholders were indirectly involved (i.e. were associated only sporadically to the intervention and/or planning activities): two were local mayors, one was working in the media and one was the regional branch of the Ministry of Social Inclusion. The remaining four stakeholders that were interviewed were not involved in the plague prevention and control activities (i.e. were not associated at any point to any of the plague prevention and control associated activities that were performed). These were: two other local mayors, the representative of the local agricultural agency and the director of the local educational center. Of the 10 additional stakeholders that should have been involved as mentioned by those initially selected, four related to civil society (local church, workers’ association, communal water association and board of water usage), 2 related to the private sector (sugarcane production company and local merchants’ association), and the remaining 4 were other sectors (agriculture, animal health, housing and police).

### Determinants of plague: Community vs. stakeholders

**Fig. 1:**
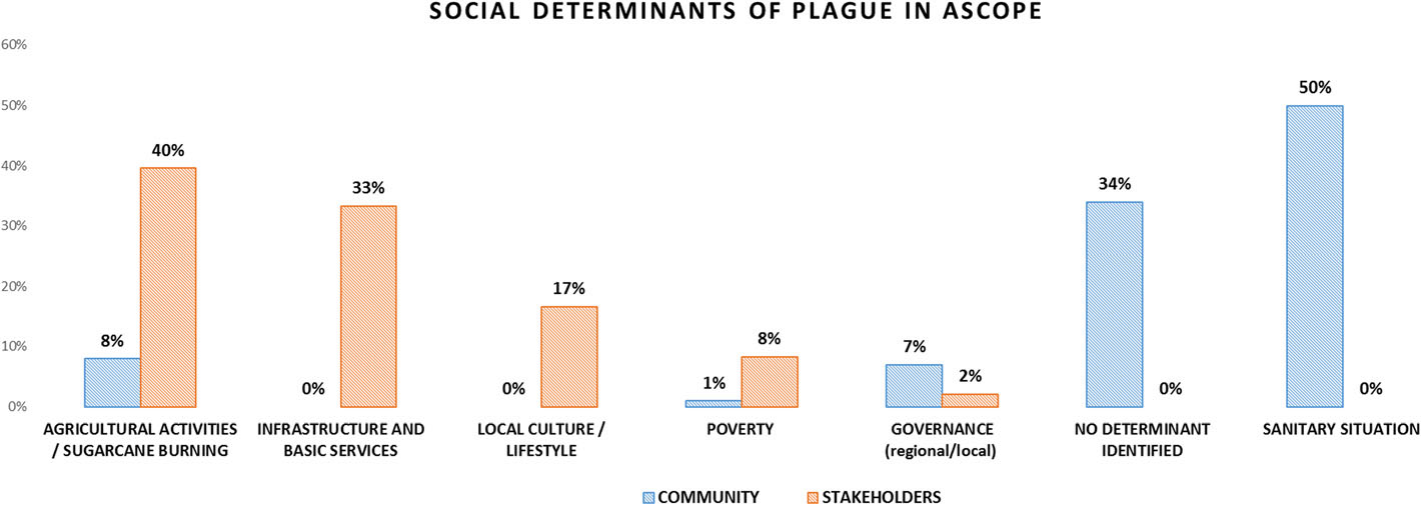
Determinants of plague: community vs. stakeholders

Comparative results between the community and the stakeholders regarding the determinants of plague are presented in Fig. 1.

The results show that the community considered the sanitary situation as the main determinant of plague whereas stakeholders point out agricultural practices such as sugarcane burning as the main root cause of plague.

### Sectors to be involved in plague prevention

**Fig. 2:**
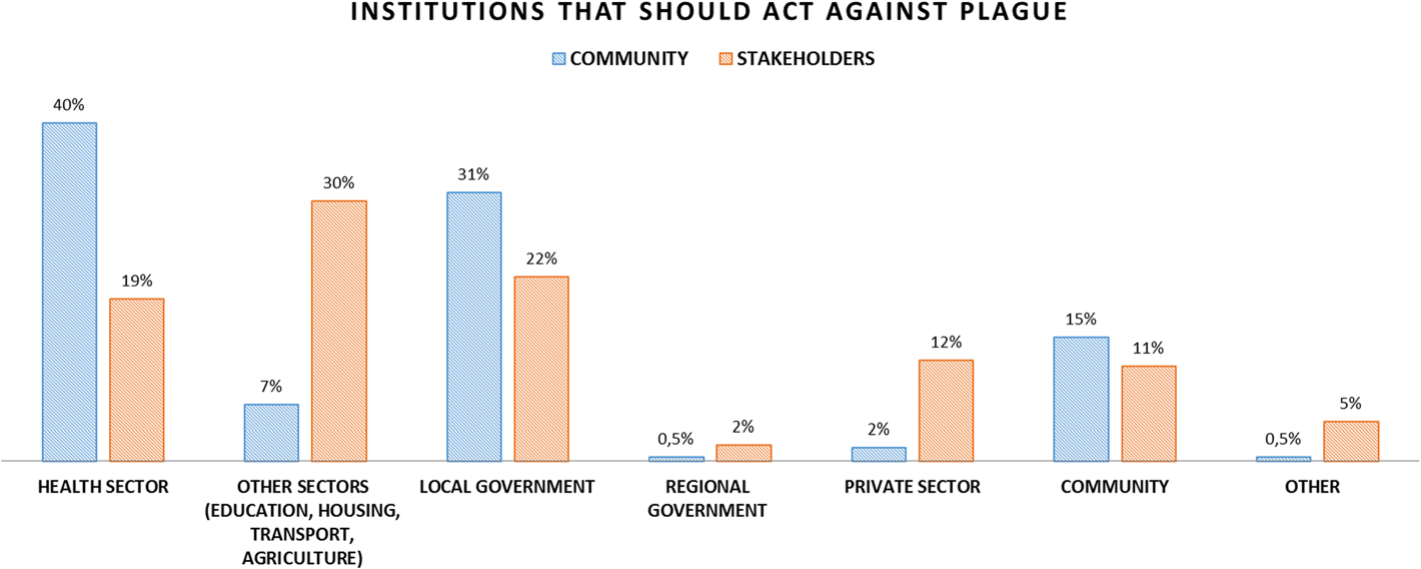
Institutions that should be involved in plague prevention: community vs. stakeholders

Community and institutional network participants were asked regarding their perceptions of what sectors should be involved in preventing plague. Results are shown in Fig. 2 below.

Results show that the community strongly thinks the health sector and the local government are the sectors to be involved in preventive actions, whereas stakeholders focus mostly on the involvement of sectors such as education, housing transport and agriculture.

### Actions to perform for plague prevention

**Fig. 3:**
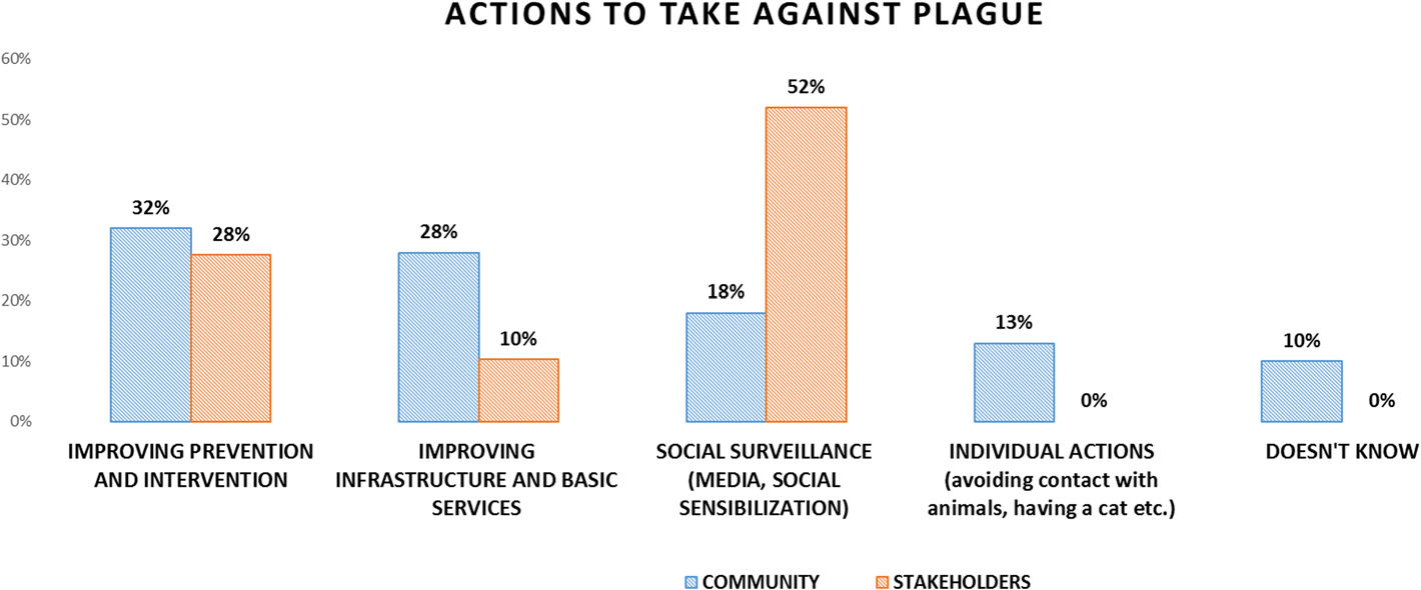
Actions suggested against the plague problem: community vs. stakeholders

Similarly, participants of both groups were asked regarding the actions that should be implemented with regards to the plague problem. Results are presented below (see Fig. 3).

Regarding the actions suggested against plague, stakeholders consider social surveillance as the most important, whereas the community highlights improving prevention and intervention activities.

## Discussion

### Determinants of plague: Local community vs. institutions involved

Results obtained from the analysis of descriptive variables depict a local community with a relatively high level of education but which lives in rather low hygienic conditions. The image provided illustrates the social and environmental determinants in which the community lives in. It depicts a community living in precarious household infrastructure conditions and physically located within an ecosystem where intensive agricultural production is ubiquitous. It has been mentioned elsewhere [44] that there is evidence of increased rodent presence when agricultural productions are introduced. Sugarcane production was introduced in Ascope at the end of the 18th century [46], it being an area classified as semi-arid [1]. Although the exact dynamics of sylvatic and peridomestic synantropic rodents has not yet been established in the context of Ascope, the household location might facilitate the interaction between rodents and humans. Additionally, the most prevalent household infrastructure materials (adobe wall and corrugate roof) are building resources which are permeable to rodents. Community members mentioned that adobe walls are easily chewed on their foundation by rodents and that the holes left on top of the walls by the corrugate roof allow rodents to enter the households by climbing the adobe walls (most generally at night). This allows rodents - attracted by stored food [47] - to easily enter the households. An additional factor that might facilitate rodent-human interaction is the agricultural practice of “slash and burn” during sugarcane harvest. Community members mentioned that they tended to see more rodents when there was sugar cane harvest (79%). Indeed sugarcane production has been linked to the presence of rodents (*Rattus rattus* and *Rattus norvegicus*) [47]. This harvesting practice might influence rodents’ ecology and dynamics, facilitating an increased interaction between rodents and humans. Finally, the virtual lack of excreta and solid waste removal in the study communities generates an ecosystem which is prone to attract rodents in search of food [47]. This might be enhanced by the presence of animals inside the household (60%), including the presence of guinea pigs (28%). Several authors have demonstrated that the presence of animals within households – including guinea pigs and cats - is positively correlated with plague occurrence [8, 9, 19, 25]. The situation depicted demonstrates the complexity, diversity and intertwined nature of root causes or determinants of plague endemicity. Indeed if each of the determinants mentioned above is not dealt with, unilateral actions from the Ministry of Health will not be enough to end plague endemicity. For example, in such conditions of infrastructure, health prevention messages propounded by the Ministry of Health and local health authorities targeting household cleanness might be difficult to implement. A wider and truthful compromise of intersectoral coordination and action, reached through a stakeholder engaging process, is likely to be more successful.

Non-surprisingly, when comparing the determinants identified by the local community, the first one ranked refers to the hygienic situation (50%). However, no other root cause could be identified by the community since 34% of the citations to the interviewed community did not identify a specific root cause. They referred exclusively to the presence of rats – which is the reservoir but not a determinant or root cause- and could not go farther in the causality thinking process. The ranking of determinants at community level diverges with that performed by the policy-makers or institutional sector. Within this group the main determinant ranked related to agricultural activities and sugarcane burning (40% of citations), followed by the lack of infrastructure and basic services (33%) and local culture or lifestyles (17%).

The differing views between the two groups show the gap existing between the community and the decision-makers. This difference demonstrates first, the poor understanding of the community regarding the disease’s determinants other than the proximate (i.e. household) hygienic situation, and second, that policy-makers are aware of the risky conditions in which the communities live, particularly regarding agricultural practices and infrastructure conditions. This observation suggests that engaging the community and policy-makers in a participatory and transdisciplinary process could contribute to a mutual understanding on the root causes of the plague endemicity and favor the identification and implementation of appropriate solutions.

### Plague risk perception levels at community level

The analysis of risk and how it is perceived at community level might help in understanding the existing gap. Following Slovic et al., risk analysis might be “dealt with in three fundamental ways. *Risk as feelings* refers to our fast, instinctive, and intuitive reactions to danger. *Risk as analysis* brings logic, reason, and scientific deliberation to bear on hazard management. When our ancient instincts and our modern scientific analyses clash, we become painfully aware of a third reality—*risk as politics”* [48]. It is clear from the descriptive data that most community members know about plague and the lethal risk it entails, which is likely to be associated with the recent experience of plague (i.e. “risk as feelings”). However more than half of the community members do not know the transmission mechanisms and more than one third are not aware of the symptoms associated with the disease (i.e. “risk as analysis”). Risk is associated with the presence of rodents but weakly to the presence of fleas. Thus, the community indeed knows about the existence of plague but basically is not able to identify or recognize the disease. This has two main implications. First, community members’ local practices in terms of rodent control might not be the most appropriate, given that they kill the rodents. When rodents are dead, fleas tend to search for other hosts. Fleas will either directly bite humans and infect them, or hop into intra-household guinea pigs (a third still has guinea pigs inside the household) infecting them. Such guinea pigs when they develop symptoms are rapidly slaughtered – not discarded - by the community members (one member is reported to have mentioned that they ate them because “the guinea pig was sad”), who get in turn infected by handling and manipulating the slaughtered sick animal [9]. Second, community members’ health seeking behavior might be impaired since they will not be able to recognize the disease and swiftly look for the nearest health care center for treatment.

In spite of the strong linkage made by community members’ between plague and rodents, a weak link is reported by the same community members between rodents and intensive agricultural practices. The low level of risk perception associated with the habitat and ecosystems’ conditions, in which the local community lives, represents an important barrier in the implementation of preventive measures against plague. These findings show that plague is still seen by the community as a medical or health problem, and is not recognized as a complex situation whereby an intertwined chain of causation – or determinants - leads to endemicity. As suggested by Weihs and Mertens (2013) [49], a participatory and transdisciplinary knowledge generation process, integrating local experience, stakeholders practices and scientific knowledge, would likely improve the systemic understanding of the multiple and interconnected causes of the health problem and more easily generate agreed upon and effective actions. The use of existing frameworks focusing on processes such as the Driving Force–Pressure–State–Exposure–Effect–Action (DPSEEA) could provide a platform for stakeholders to engage and commit on an action roadmap.

### Actions to be undertaken and sectors that should be involved: Community vs. decision makers

The third type of risk mentioned above - risk as politics – is clearly depicted in this section. Despite policy makers having ranked agricultural production and infrastructure and basic services as the first two determinants of plague in Ascope, it is surprising to observe that the main actions proposed relate to social surveillance and prevention and intervention activities. In addition, besides having ranked agricultural practices as the main determinant of plague in the case of Ascope, it is unexpected to see that actions involving private sector sugarcane companies only rank fourth. Thus, as stated by Slovic, there is a clash between the scientific analysis performed and the proposed solutions by policy makers. On the community members’ side there seems to be coherence between the determinants identified (i.e. sanitary conditions), the actions proposed (i.e. prevention and intervention activities, and infrastructure and basic services) and the institutions that should be involved (i.e. health sector and local government). Given that the community does not link plague to agricultural practices it is not surprising that the sugarcane production companies are listed last.

Stakeholders’ discourse shows a level of inconsistency regarding the actions to be implemented and the sectors to involve in solving the determinants of plague. Particularly interesting is stakeholders’ consideration of social surveillance as one of the main actions to be implemented, whereas the community is not able to correctly identify plague. In addition, what are the underlying reasons why agricultural production is identified as the main root cause of plague but sugarcane companies are lightly mentioned (12%) in solving the problem? Why is it that having identified infrastructure and basic services as the second root cause of plague in Ascope, the actions proposed associated to the same category only rank third (10%)?

## Conclusion

The study in Ascope depicts a situation in which local community members live in a precarious infrastructural and sanitary setting but are aware of such limitations and ask for solutions geared towards these problems. However, they have low risk perception levels given that root causes of plague are not identified nor transmission mechanisms or symptoms. Following the results of the study, at community level efforts should focus on: (i) targeting health education messages to facilitate understanding of the disease and thus prevention and early reaction, (ii) improving household infrastructure, (iii) fostering the implementation of water and sanitation, (iv) promoting solid waste removal, and (v) promoting appropriate handling and tenancy of domestic animals.

The more convoluted scenario depicted by the stakeholders’ group shows, that inconsistencies between perceived causes and suggested solutions, might be associated to lack of communication and collaboration across stakeholders’ categories and sectors of action. The relation between the stakeholders’ group and the different sectors that were mentioned as needed in problem-solving – particularly the sugarcane production company - but that were not present in the response might need to be further examined. Following the study results, at stakeholders’ level efforts should focus on (i) promoting multisectoral policies and plan of actions lead by local mayors integrating public, private and civil society sectors from a participatory process perspective to enable early engagement of all stakeholders, (ii) adapting the legal and regulatory framework to allow or facilitate the implementation and enforcing of norms providing incentives for multisectoral approach for local community improvement, and (iii) promoting healthy housing and healthy community.

Finally, there is a need (i) to better understand rodent biology and dynamics associated to sugarcane production and “slash and burn” harvesting techniques, (ii) to systematically integrate and map biological surveillance indicators with socio-ecological determinants indicators for early warning, and (iii) to perform a deeper analysis of the strength, coherence and cooperation level of the stakeholder response network to further understand the associated gaps identified.

## Abbreviations

CBM: Community-Based Management
ENSO: El Niño Southern Oscillation
INS: “Instituto Nacional de Salud” (National Institute of Health)
PAHO: Pan-American Health Organization
